# Longitudinal Comparison of Calprotectin and C-Reactive Protein in Rheumatoid Arthritis: Real-World Evidence Across Three Targeted Therapies

**DOI:** 10.3390/diagnostics16010064

**Published:** 2025-12-24

**Authors:** Angelo Fassio, Isotta Galvagni, Silvia Sartoris, Denise Alessandra Cassandrini, Federico Aldegheri, Maurizio Rossini, Francesco Pollastri, Giovanni Adami, Davide Gatti, Rosanna Somma, Matteo Appoloni, Antonio Carletto, Vincenzo Bronte, Alessandra Arcolaci

**Affiliations:** 1 Rheumatology Unit, University of Verona, 37126 Verona, Italy; angelo.fassio@aovr.veneto.it (A.F.); isottagalvagni8@gmail.com (I.G.); maurizio.rossini@univr.it (M.R.); franpollastri@yahoo.it (F.P.); giovanni.adami@univr.it (G.A.); davide.gatti@univr.it (D.G.); rosanna.somma20@gmail.com (R.S.); appolonimatteo.95@gmail.com (M.A.); antonio.carletto@aovr.veneto.it (A.C.); 2 Immunology Unit, University Hospital of Verona, 37126 Verona, Italy; silvia.sartoris@univr.it (S.S.); denisealessandra.cassandrini@aovr.veneto.it (D.A.C.); vincenzo.bronte@univr.it (V.B.); alessandra.arcolaci@aovr.veneto.it (A.A.); 3 Immunology Section, Department of Medicine, University of Verona, 37126 Verona, Italy

**Keywords:** calprotectin, rheumatoid arthritis, biological disease-modifying antirheumatic drugs, C-reactive protein

## Abstract

**Background/Objectives**: Serum calprotectin is a promising biomarker of inflammation in rheumatoid arthritis (RA), yet real-world longitudinal comparisons across different targeted therapies remain limited. We aimed to evaluate the dynamics and remission-predictive ability of serum calprotectin and C-reactive protein (CRP) in RA patients treated with adalimumab, upadacitinib, or tocilizumab. **Methods:** In this retrospective cohort study, patients with RA initiating one of the above therapies were included. Serum calprotectin and CRP were measured at baseline, month 3, and month 6. Disease activity was assessed by DAS28 and Clinical Disease Activity Index (CDAI). Linear mixed-effects models adjusted for cumulative prednisone dose were used to assess biomarker trends over time. ROC curve analyses based on CDAI remission (≤2.8) evaluated the discriminative performance of calprotectin and CRP, stratified by treatment subgroups. **Results:** Sixty patients were enrolled (20 receiving tocilizumab, 20 adalimumab and 20 upadacitinib). Significant reductions in serum calprotectin, CRP, and DAS28 were observed over time (*p* < 0.001 for all), independent of treatment group. In the overall cohort including baseline, CRP outperformed calprotectin (AUC 0.739 vs. 0.636; *p* = 0.044). Among patients treated with adalimumab or upadacitinib, calprotectin significantly outperformed CRP (AUC 0.929 vs. 0.857; *p* = 0.049). In the tocilizumab group, both biomarkers showed similar AUCs (*p* = 0.888). **Conclusions**: Serum calprotectin declined significantly after treatment initiation and outperformed CRP in identifying remission under TNFα and JAK inhibition. It also retained a good performance under IL-6 blockade. These findings support its role as a treatment-sensitive biomarker suggesting a complementary role alongside CRP in RA monitoring, particularly in settings where CRP reliability is pharmacologically suppressed.

## 1. Introduction

Rheumatoid arthritis (RA) is a chronic inflammatory disease characterized by synovial proliferation, systemic inflammation, and progressive joint damage [1]. Monitoring disease activity is a cornerstone of RA management, guiding treatment decisions and the achievement of remission or low disease activity targets. Currently, composite indices such as the Disease Activity Score-28 (DAS28) or Clinical Disease Activity Index (CDAI), in combination with acute-phase reactants like C-reactive protein (CRP), are routinely used to assess response to therapy [2,3]. However, the reliability of CRP as a biomarker is increasingly challenged by the use of interleukin-6 (IL-6) inhibitors, which can suppress hepatic CRP production independently of disease activity [4].

Calprotectin, a heterodimer of the calcium-binding proteins S100A8/S100A9, is abundantly expressed in neutrophils and, to a lesser extent, in monocytes and macrophages, and is released upon activation of these phagocytic cells during inflammatory responses [1]. Consequently, circulating calprotectin levels closely reflect myeloid cell-driven inflammation at the tissue level. In RA calprotectin has emerged as a promising serum biomarker of inflammation, showing a strong correlation with ultrasound-detected synovitis and treatment response, even in patients with normal CRP levels [5,6,7,8,9]. Several cross-sectional and longitudinal studies have suggested that serum calprotectin levels decrease in response to effective therapy and may predict disease flare or persistence of subclinical synovitis [10,11,12,13].

Yet, despite these encouraging findings, calprotectin remains underused in clinical practice, partly due to the limited availability of comparative, real-world longitudinal data across different RA treatment classes, such as inhibitors of tumor necrosis factor-α (TNF-α), Janus kinases (JAK), and interleukin-6 (IL-6). In particular, whether calprotectin provides added value over CRP in identifying remission across biologic agents, depending on each drug’s mechanism of action, has not been definitively established [14].

To address this gap, we conducted a longitudinal evaluation of serum calprotectin, CRP, DAS28 and CDAI, in patients with RA initiating treatment with adalimumab (anti-TNF-α fully human monoclonal antibody), upadacitinib (JAK inhibitor), or tocilizumab (anti-IL-6 humanized monoclonal antibody), aiming to assess their ability to discriminate clinical remission. By analyzing changes over time and treatment-specific differences through longitudinal modeling and ROC analysis, we sought to investigate whether serum calprotectin provides mechanistically sensitive and treatment-responsive information that could support more tailored monitoring strategies.

Despite promising data, calprotectin has not yet been integrated into routine RA monitoring. Understanding whether its performance varies depending on treatment mechanism may open the way to more personalized biomarker-based strategies. We therefore aimed not only to evaluate its longitudinal behavior, but also to assess its comparative advantage across treatment classes, in real-world clinical conditions.

## 2. Materials and Methods

### 2.1. Study Design and Settings

We conducted a retrospective study based on longitudinal data from patients with RA treated with adalimumab, tocilizumab, or upadacitinib. The study was carried out at the Rheumatology Clinic and at the Immunology Unit of the University Hospital of Verona (Verona, Italy).

### 2.2. Participants and Data Collection

Eligible adults (≥18 years) with RA, diagnosed according to the 2010 ACR/EULAR classification criteria, who had initiated treatment with adalimumab, tocilizumab, or upadacitinib in routine clinical practice, were retrospectively identified from electronic medical records. Patients were classified into three treatment groups based on the biologic or targeted synthetic DMARD they were receiving at the time of data collection (adalimumab n = 20, tocilizumab n = 20, upadacitinib n = 20).

Exclusion criteria were lack of signed informed consent, active malignancy, ongoing infections during the observation period, and the presence of significant renal, hepatic, endocrine, cardiac, or metabolic bone disorders.

Data on patient demographics, clinical history, CRP values, RA characteristics, DAS28 scores, disease remission according to CDAI, and pharmacological treatment were extracted from medical records at baseline, and at three and six months following therapy initiation. The cumulative prednisone dose administered during the study period was estimated from clinicians’ reports in electronic medical records. Importantly, remission was defined using CDAI criteria, which do not include CRP or ESR, thereby avoiding circularity in the ROC analysis of inflammatory biomarkers.

### 2.3. Serum Calprotectin Measurement

Serum samples were collected at baseline and at month 3 and 6, in the morning following an overnight fast. The samples were stored at −50 °C until analysis. Calprotectin levels were assessed in a single batch using the QUANTA Flash Circulating Calprotectin chemiluminescent immunoassay (CIA) (Werfen Inova Diagnostics, Milan, Italy) on the BIO-FLASH system, according to the manufacturer’s instructions. A cut-off of ≥2.00 μg/mL was used to define a positive result, with normal serum calprotectin levels considered to be below this value.

### 2.4. Statistical Analysis

Between-group differences at baseline were assessed using one-way ANOVA or the Kruskal–Wallis test for continuous variables, and the Chi-square test for categorical variables.

We performed an exploratory longitudinal analysis to assess changes over time in serum calprotectin, CRP, and DAS28 across three timepoints (baseline, month 3, and month 6). To adjust for concomitant glucocorticoid exposure, the cumulative prednisone dose administered during the study period was included as a covariate in all models.

To improve the normality of residuals and homoscedasticity, serum calprotectin and CRP values were log-transformed prior to analysis. Repeated measures within subjects were modeled using linear mixed-effects models with a random intercept for patient ID. We compared models including and excluding the interaction between time and treatment using the Akaike Information Criterion (AIC) and Bayes factors to assess model fit and parsimony.

Final models incorporated time as a categorical fixed effect (baseline, month 3, month 6), with or without adjustment for cumulative prednisone exposure.

To assess the discriminative ability of calprotectin and CRP for predicting disease activity, receiver operating characteristic (ROC) curve analyses were performed using CDAI remission (CDAI ≤ 2.8) as the binary outcome. Importantly, CDAI remission was chosen as the reference outcome because it does not incorporate acute-phase reactants. This avoids circularity between biomarker levels and the definition of remission, ensuring that neither CRP nor calprotectin can drive the outcome classification. Consequently, differences in serological profiles (e.g., ACPA status) are unlikely to affect the comparative performance of the biomarkers. Logistic mixed-effects models with a random intercept for patient ID were fitted separately for calprotectin and CRP using a binomial distribution. Predicted probabilities were used to generate ROC curves. Analyses were performed on the overall sample (with and without baseline) and stratified by treatment group (tocilizumab vs. adalimumab/upadacitinib, excluding baseline). The area under the curve (AUC) was calculated for each marker, and AUCs were compared using DeLong’s test.

All analyses were conducted in R (v. 4.3.1). Model residuals were visually inspected to verify assumptions of distributional symmetry and variance homogeneity. Two-sided *p*-values of 0.05 or less were considered statistically significant.

### 2.5. Ethical Approval

The study was approved by the local Ethics Committee (protocol 1483 CESC) and conducted in accordance with the 1964 Declaration of Helsinki and its later amendments or comparable ethical standards. Written informed consent was obtained from all participants.

## 3. Results

A total of 60 patients were included, contributing 180 observations across three treatment arms. Baseline characteristics of the overall sample and proportion of subjects in remission according to CDAI at baseline, month 3 and 6 are summarized in Table 1. Groups were generally comparable except for cumulative prednisone exposure, which was significantly higher (*p* < 0.001) in the tocilizumab arm with respect to adalimumab and upadacitinib.

**Figure 1:**
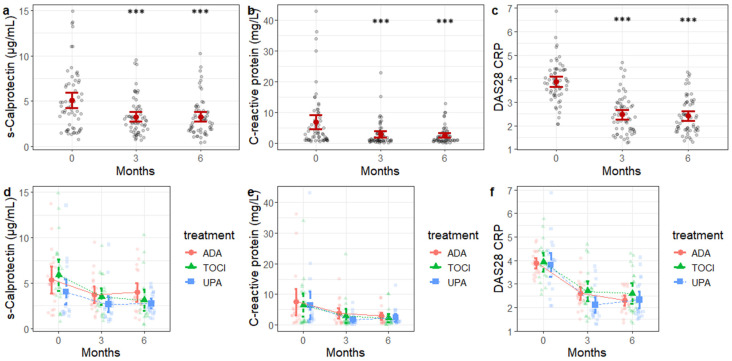
Trends over time for serum calprotectin, C-reactive protein and DAS28 CRP for the overall sample (panel (**a**–**c**)), and according to treatment subgroups (panel (**d**–**f**)). Grey dots and light-colored symbols represent individual patient values; solid colored markers with error bars indicate group means (±standard error). ADA, adalimumab; TOCI, tocilizumab, UPA, upadacitinib. *** *p* < 0.001 versus baseline.

After adjustment for cumulative prednisone dose, a significant reduction over time was observed for all three variables (Figure 1a–c). Serum calprotectin decreased significantly at month 3 (*p* = 0.0002) and further at month 6 (*p* < 0.001) compared to baseline. CRP values declined significantly at month 3 and month 6 (*p* < 0.001 for both comparisons). Similarly, DAS28 scores showed a marked and highly significant reduction at both follow-up visits (*p* < 0.001 for both timepoints). No significant between-group (treatment) differences were observed for calprotectin, CRP or DAS28 (Figure 1d–f).

**Figure 2:**
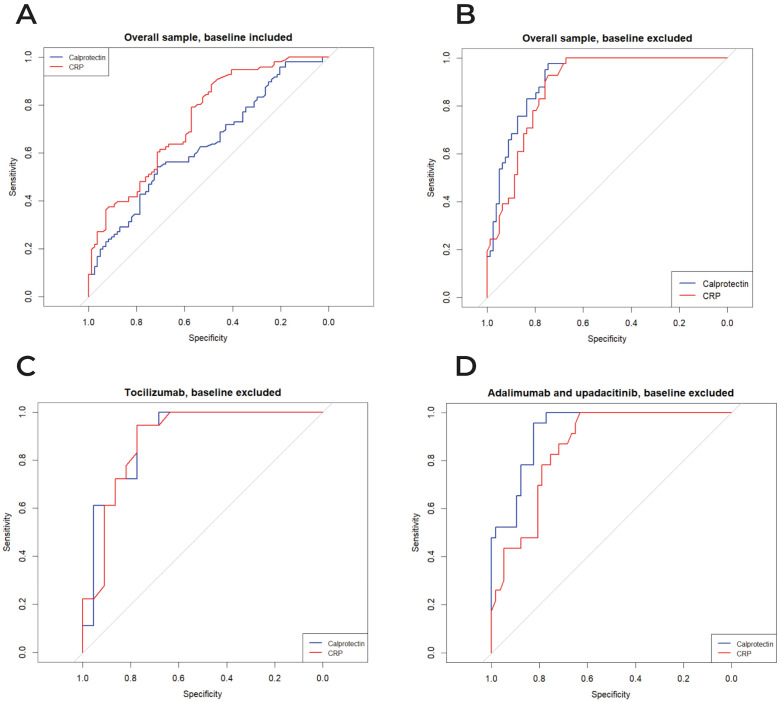
ROC curves comparing the diagnostic performance of serum calprotectin and CRP for CDAI remission, for the overall sample, baseline included (panel **A**) and excluded (panel **B**), tocilizumab (panel **C**), and adalimumab/upadacitinib (panel **D**). The grey 45° line indicates the no-discrimination reference.

In the full dataset including baseline observations, the discriminative performance of calprotectin and CRP for identifying patients in CDAI remission was modest, with AUCs of 0.636 and 0.739, respectively (*p* = 0.044; Figure 2A). When the analysis was restricted to follow-up data only (baseline excluded), calprotectin showed an AUC of 0.911, and CRP an AUC of 0.880, with no statistically significant difference between them (*p* = 0.302; Figure 2B). In the subgroup of patients treated with tocilizumab (baseline excluded), calprotectin and CRP performed similarly, with AUCs of 0.894 and 0.885, respectively (*p* = 0.888; Figure 2C). Conversely, in patients treated with adalimumab or upadacitinib (baseline excluded), calprotectin demonstrated significantly better discrimination than CRP, with AUCs of 0.929 and 0.857, respectively (*p* = 0.049; Figure 2D).

## 4. Discussion

In this real-world exploratory longitudinal study, we evaluated the dynamics of serum calprotectin, CRP, and DAS28 over a six-month period in RA patients treated with adalimumab, upadacitinib, or tocilizumab. To our knowledge, this is the first comparative longitudinal analysis evaluating calprotectin performance across targeted therapies with distinct mechanisms, using CDAI remission as a validated CRP-independent outcome. All three markers declined significantly over time, independently of cumulative glucocorticoid exposure. While time exerted a consistent effect on biomarker levels and clinical disease activity, notably, the absence of a significant interaction between treatment and time suggests a common temporal pattern of response across therapies. However, the small sample may have limited the statistical power to detect moderate differences.

Serum calprotectin, a heterodimeric protein released by activated neutrophils and monocytes, has emerged as a promising biomarker of disease activity in RA, particularly in relation to subclinical synovitis detected by ultrasound [15]. Baseline levels in our cohort were within the range previously reported for active RA and consistently higher than those described in non-inflammatory or healthy subjects [16,17]. In line with these observations, our study showed a marked decline in calprotectin levels as early as month 3, followed by a further reduction at month 6. Similar trends were observed for CRP and DAS28, supporting the utility of calprotectin, as acute-phase reactants, in tracking disease activity and, thus, treatment response, after biologic or targeted synthetic disease-modifying anti-rheumatic drugs (DMARDs) initiation.

Interestingly, our ROC analyses provide additional insights into the comparative performance of calprotectin and CRP as biomarkers of remission. In the full dataset including baseline, both markers showed only modest discriminative performance for CDAI remission, with CRP performing slightly better (AUC 0.739 vs. 0.636; *p* = 0.044). However, upon exclusion of baseline data, thus restricting the analysis to patients already under active treatment, calprotectin’s performance improved markedly, compared to CRP (AUC 0.911 vs. 0.880), although this difference did not reach statistical significance (*p* = 0.302). This discrepancy suggests that calprotectin’s diagnostic role may be enhanced under treatment, potentially reflecting a reduced variability due to inflammation-related confounders present at baseline. Indeed, baseline calprotectin levels are highly heterogeneous, possibly influenced by variable pre-treatment inflammatory burden, comorbidities [18], or non-synovial sources of neutrophil activation, like metabolic stress or other systemic triggers [19]. Under treatment, particularly in patients approaching low disease activity, calprotectin may more specifically reflect residual synovial inflammation, while CRP may remain more susceptible to pharmacologic suppression.

Alternatively, the lower AUC at baseline may stem from non-linear or threshold-based dynamics in calprotectin’s relationship with remission status, which could be masked in early disease phases or before therapeutic response is established. These findings underscore the importance of timing when interpreting biomarker performance and support the concept that the diagnostic and prognostic utility of calprotectin may be most informative after treatment initiation. Recent evidence have similarly shown that a single baseline calprotectin measurement fails to predict outcomes, highlighting the need for longitudinal assessment under active therapy [20]. Stratified analyses by treatment group provided further nuance.

In our tocilizumab-treated subgroup, ROC curve analyses showed virtually identical AUCs for calprotectin and CRP in detecting CDAI remission (0.894 vs. 0.885; *p* = 0.888). These findings were somewhat unexpected, given the well-documented pharmacologic suppression of CRP under IL-6 inhibition (19), raising concerns about its reliability as a remission marker. At first glance, this appears, for instance, inconsistent with findings by Gernert et al. [21], who reported poor CRP performance in patients receiving IL-6 inhibitors. However, important methodological differences may account for this. Whereas Gernert et al. conducted a cross-sectional analysis including patients treated for at least three months, our study assessed biomarker performance including the data at 3 and 6 months after treatment initiation. This temporal alignment likely minimized heterogeneity in treatment exposure and biomarker kinetics, potentially explaining the similar AUCs we observed. Additionally, in the earlier phases of IL-6 inhibition, CRP suppression may not be complete, allowing some residual correlation with disease activity. Furthermore, the significant therapeutic effectiveness of the included b/tsDMARDs, with a high remission rate by month 6, may have introduced a “floor effect”, eroding the disease activity variability and limiting the ability to detect subtle differences between biomarkers. This effect may have been amplified by the higher cumulative glucocorticoid exposure in the tocilizumab group: corticosteroids upregulate S100A8/A9 transcription and stabilize its mRNA via IL-10 and Mitogen-Activated Protein Kinase (MAPK) signaling, promoting sustained calprotectin production even in the absence of active synovitis [22]. Finally, the relatively small sample size of this subgroup may have further limited statistical power. Future studies with larger cohorts and imaging-based disease activity measures are warranted to clarify whether calprotectin consistently outperforms CRP under IL-6 blockade.

Conversely, among patients treated with adalimumab or upadacitinib, where the cumulative steroid dose was significantly lower, calprotectin demonstrated superior discriminative capacity (AUC 0.929 vs. 0.857; *p* = 0.049), in line with recent head-to-head comparisons suggesting that calprotectin may better reflect residual inflammation in patients treated with JAK inhibitors or TNF blockers [23,24].

Our findings reinforce and suggests that calprotectin may be particularly valuable in therapeutic contexts where CRP remains pharmacologically modifiable. Collectively, our findings support calprotectin as a robust biomarker of RA disease activity. They also align with recent meta-analyses indicating that serum calprotectin is associated with treatment response and subclinical synovitis, and may serve as a surrogate endpoint in clinical trials [25]. From a clinical perspective, our findings further clarify the complementary roles of CRP and calprotectin in RA monitoring. CRP remains a widely available marker of systemic inflammation, but its interpretability is limited in therapeutic contexts where hepatic CRP synthesis is pharmacologically suppressed, such as IL-6 inhibition. In contrast, calprotectin reflects activation of neutrophils and monocytes within the inflamed synovium and therefore remains sensitive to residual disease activity even when CRP levels are low because of treatment. This mechanistic distinction becomes particularly relevant once patients move toward low disease activity, a phase in which calprotectin may more accurately capture residual synovial inflammation while CRP becomes increasingly susceptible to pharmacologic modulation. Together, these observations support the integration of calprotectin as an adjunct biomarker during follow-up, especially in patients receiving therapies that directly modulate acute-phase reactants.

This study has several strengths. First, it provides a longitudinal comparison of serum calprotectin and CRP, across three clinically relevant timepoints, in patients with RA treated with distinct targeted therapies (IL-6 receptor inhibitor, TNFα inhibitor, and JAK inhibitor). Moreover, the use of CDAI as a composite CRP-independent outcome, enhances the clinical interpretability of the findings Mixed-effects models adjusted for cumulative glucocorticoid exposure, addressed within-subject variability and the potential confounding effect of concomitant corticosteroids. Furthermore, the stratified ROC analysis highlights mechanistic differences in biomarker performance across therapeutic classes, supporting a tailored approach to biomarker interpretation.

However, several limitations must be acknowledged. First, the overall sample size restricted the statistical power of stratified evaluations, particularly in the subgroup treated with tocilizumab, where both biomarker kinetics and CRP suppression may differ mechanistically from other therapies. This limited our ability to detect small-to-moderate treatment-specific differences or to fully characterize biomarker performance across subgroups. Second, although cumulative prednisone dose was included as an adjustment variable, other potential confounders such as disease duration, prior biologic exposure, baseline disease activity, comorbidities, and serologic status may have influenced biomarker levels or clinical response. ACPA was evenly distributed across treatment groups and showed no association with baseline calprotectin or CRP values in our cohort; however, the sample size was insufficient to support robust antibody-stratified analyses. Third, the retrospective study design, with follow-up limited to routine clinical visits at months three and six, may have introduced heterogeneity in glucocorticoid tapering, treatment adherence, or timing of biomarker assessment. Prospective studies with standardized follow-up intervals and protocolized sampling are warranted to confirm these findings. Fourth, all data were derived from a single center, which may limit external generalizability. Although both biomarkers showed consistent longitudinal behavior, their performance might differ in populations with varying disease severity, comorbid metabolic inflammation, or different patterns of glucocorticoid use. Finally, ROC curve analyses were based on mixed-effects logistic models that were not cross-validated, raising the possible issue of overfitting, particularly in subgroup analyses. External replication and validation with imaging-based endpoints (e.g., ultrasound or MRI synovitis) will be essential to confirm the comparative utility of calprotectin and CRP in longitudinal RA monitoring.

## 5. Conclusions

This real-world longitudinal study demonstrates that serum calprotectin declined significantly over time in RA patients initiating treatment with adalimumab, upadacitinib, or tocilizumab, mirroring changes in CRP and DAS28. While CRP remains a widely used biomarker, calprotectin demonstrated a superior discriminative ability for clinical remission in patients receiving TNFα or JAK inhibitors and maintained reliable performance even under IL-6 blockade. These results reinforce the role of calprotectin as a treatment-responsive biomarker and support its integration into longitudinal monitoring of RA, offering additive value to CRP, particularly in settings where the latter may be unreliable due to pharmacological suppression. Further prospective studies incorporating imaging endpoints are warranted to confirm its role as a mechanistically sensitive and treatment-responsive biomarker and to validate its use in routine monitoring and clinical decision-making.

## Figures and Tables

**Table 1:** Baseline characteristics of the overall sample and proportion of subject in remission according to CDAI at baseline, month 3 and month 6. Data are expressed as mean (standard deviation), median [interquartile range] or absolute number (percentage), as appropriate. * *p* < 0.01 versus adalimumab and upadacitinib.

	Adalimumab n = 20	Tocilizumab n = 20	Upadacitinib n = 20	*p*-Value
M:F	6/14	8/12	3/17	0.29
Age (years)	62.20 (14.75)	66.85 (9.05)	59.05 (9.34)	0.10
Disease duration (years)	14.60 [5.75;13]	19.25 [7;16]	12.10 [8–15.3]	0.14
BMI (kg/m^2^)	25.80 (3.89)	27.08 (5.01)	26.51 (6.32)	0.84
Baseline s-Calprotectin (ug/mL)	4.91 [2.8;7.13]	4.73 [3.57;8.12]	3.47 [1.85;5.19]	0.22
Baseline CRP (mg/L)	3.9 [2.6;7.65]	1.7 [1;10.7]	2.45 [1.28;5.25]	0.56
Baseline DAS28	3.88 (0.50)	3.92 (0.88)	3.82 (1.13)	0.92
ACPA positivity	9 (45.0)	13 (65.0)	11 (55.0)	0.44
Rheumatoid factor positivity	13 (65.0)	11 (55.0)	10 (50.0)	0.62
Erosive disease	10 (50.0)	7 (35.0)	8 (40.0)	0.61
Cumulative prednisone (mg)	225 [0.0;570]	945 [474;1215] *	348 [116;631]	<0.001
CDAI remission, baseline	0 (0)	1 (5)	4 (20)	0.06
CDAI remission, month 3	12 (60)	10 (50)	16 (80)	0.13
CDAI remission, month 6	15 (75)	12 (60)	14 (70)	0.58

For each outcome, models including the interaction term between time and treatment were compared to reduced models without interaction. In all cases—serum calprotectin, CRP, and DAS28—the inclusion of the interaction term did not improve model fit and was excluded from the final analyses.

## Data Availability

Data cannot be publicly shared due to privacy and ethical restrictions. Data of the analysis is available upon reasonable request.

## Abbreviations

The following abbreviations are used in this manuscript:

ACPA	Anti-Citrullinated Protein Antibodies
ADA	Adalimumab
AIC	Akaike Information Criterion
ANOVA	Analysis of Variance
AUC	Area Under the Curve
bDMARDs	Biological Disease-Modifying Anti-Rheumatic Drugs
BMI	Body Mass Index
CDAI	Clinical Disease Activity Index
CIA	Chemiluminescent Immunoassay
CRP	C-Reactive Protein
DAS28	Disease Activity Score-28
DMARDs	Disease-Modifying Anti-Rheumatic Drugs
EULAR	European Alliance of Associations for Rheumatology
IL-6	Interleukin-6
JAK	Janus Kinase
MAPK	Mitogen-Activated Protein Kinase
RA	Rheumatoid Arthritis
RF	Rheumatoid Factor
ROC	Receiver Operating Characteristic
S100A8/S100A	Calcium-binding proteins A8 and A9 (Calprotectin subunits)
SE	Standard Error
TNF-α	Tumor Necrosis Factor-alpha
TOCI	Tocilizumab
UPA	Upadacitinib
